# Neural Correlates of Conscious Motion Perception

**DOI:** 10.3389/fnhum.2018.00355

**Published:** 2018-09-10

**Authors:** Gonzalo Boncompte, Diego Cosmelli

**Affiliations:** ^1^Laboratorio de Psicofisiología, Escuela de Psicología, Pontificia Universidad Católica de Chile, Santiago, Chile; ^2^Centro Interdisciplinario de Neurociencias, Pontificia Universidad Católica de Chile, Santiago, Chile

**Keywords:** neural correlates of consciousness (NCC), consciousness, conscious perception, motion perception, perceptual feature, P3b

## Abstract

The nature of the proper neural signature of conscious perception remains a topic of active debate. Theoretical support from integrative theories of consciousness is consistent with such signature being P3b, one of the main candidates in the literature. Recent work has also put forward a mid-latency and more localized component, the Visual Awareness Negativity (VAN), as a proper Neural Correlate of Consciousness (NCC). Early local components like P1 have also been proposed. However, experiments exploring visual NCCs are conducted almost exclusively using static images as the content to be consciously perceived, favoring ventral stream processing, therefore limiting the scope of the NCCs that have been identified. Here we explored the visual NCCs isolating local motion, a dorsally processed feature, as the primary feature being consciously perceived. Physical equality between Seen and Unseen conditions in addition to a minimal contrast difference between target and no-target displays was employed. In agreement with previous literature, we found a P3b with a wide centro-parietal distribution that strongly correlated with the detection of the stimuli. P3b magnitude was larger for Seen vs. Unseen conditions, a result that was consistently observed at the single subject level. In contrast, we were unable to detect VAN in our data, regardless of whether the subject perceived or not the stimuli. In the 200–300 ms time window we found a N2pc component, consistent with the high attentional demands of our task. Early components like P1 were not observed in our data, in agreement with their proposed role in the processing of visual features, but not as proper NCCs. Our results extend the role of P3b as a content independent NCC to conscious visual motion perception.

## Introduction

Two influential theories of consciousness, Global Neuronal Workspace Theory (Baars, 1994; Sergent and Dehaene, 2004; Dehaene et al., 2014) and Integrated Information theory (Tononi, 2008, 2012), emphasize integrative neural activity as a crucial aspect of NCCs. In electroencephalographic (EEG) recordings, such integrative neural activity is consistent with observed differences in event-related potential (ERP) components like the P3b (Hillyard et al., 1971; Sergent et al., 2005; Del Cul et al., 2007; Salti et al., 2012). This is because these components are thought to be produced by widespread neural activation across different associative cortices (Bledowski et al., 2004). This contrasts with early components like P1, which is locally generated by specific areas of the extrastriatal visual cortex (Di Russo et al., 2001; Pourtois et al., 2005) and that has also been proposed as NCCs (Pins and Ffytche, 2003; Roeber et al., 2008). A mid-latency (200–300 ms) component, the Visual Awareness Negativity (VAN), has also been consistently reported as an ERP NCC (Koivisto et al., 2008; Pitts et al., 2011; Rutiku et al., 2016). Usually conscious perception modulates both VAN and P3 concomitantly (Pitts et al., 2014). However, consensus regarding which one(s) comply with the requirements of a proper-NCC (Koivisto and Revonsuo, 2010; Melloni et al., 2011; Aru et al., 2012; De Graaf et al., 2012), but also those of a content-independent NCC, remains elusive (Aru et al., 2012; De Graaf et al., 2012; de Graaf and Sack, 2015).

A notable aspect of the literature on this topic is the great diversity of contents of the target stimuli used. These include faces and houses (Tong et al., 1998), letters (Kranczioch et al., 2005), complex textures, (Supèr et al., 2001), and everyday pictures (Fernandez-duque et al., 2003), among others (Kim and Blake, 2005). The ERP NCCs evoked by these various objects are usually compared among each other without further consideration that they could be content-specific NCCs, that is neural activity that correlates with conscious perception only of a particular type of content. This has been suggested for the case of faces and the N170 component (Navajas et al., 2013; Shafto and Pitts, 2015). A recent article by Rutiku et al. (2016) addressed the possible consequences of this issue, arguing that “If experiments employ restricted categories of stimuli it is hard to tell whether the resulting NCCs are marker of only one category or whether they can be generalized to other categories as well.” They studied ERP NCCs evoked by a randomized sequence of numerous different images, thus rendering the contribution of any particular content on these images supposedly irrelevant for the grand average NCC produced. They concomitantly reported VAN and P3b as content-independent or general ERP NCCs.

Despite the fact that numerous perceptual contents can be evoked by different static images, this type of stimulus is always constituted by static shape information: spatial patterns of luminance establish contours and shapes, which in turn define the content of the image. In fact, it could be argued that the majority of stimuli used in modern studies of visual NCCs are formed by static patterns of luminance (but see for example Niedeggen et al., 2012). This type of information strongly coincides with receptive fields of the ventral visual stream (Wang et al., 1999; Doniger et al., 2000; James et al., 2003; Connor et al., 2007). Examples include numbers (Shum et al., 2013; Grotheer et al., 2016), letters (Vinckier et al., 2007; Hannagan et al., 2015), and faces (Kanwisher et al., 1997), all of them extensively used in the study of visual NCCs (Del Cul et al., 2007; Melloni et al., 2011; Pitts et al., 2014). Here we attempt to explore the ERP NCCs produced by local visual motion, which is predominantly processed by the dorsal visual stream (Goodale and Milner, 1992; Milner and Goodale, 2008). Cortical activation related to movement in the visual field initially arises in V1, however it is segregated for its processing into dorsal areas like the MT complex (Tootell et al., 1995; Andersen, 1997; Culham et al., 2001). Here we constructed a stimulation paradigm in which subjects consciously perceived the brief (100 ms) movement of a small dot with blurred edges, which could be presented either to the left or right of a central fixation point. Distractors were added in the form of dots that were identical to the target, but that did not move and instead continuously flickered across the screen during the duration of the trial. The continuous presentation of irrelevant stimuli (distractors) that shared a great number of features with the target thus impaired its visibility in a proportion of trials (for a related effect see Sahraie et al., 2001; Niedeggen et al., 2015). In this way we isolated movement as the perceptual feature to be detected while at the same time minimized the on-screen contrast differences when comparing distracter alone vs distracter plus target presentation (≈200 vs. ≈201 dots on screen, respectively, see below). The number of distractors was calibrated to ensure the detectability of the target in ~50% of the trials, without modifying the contrast or duration of the target stimulus. We explore the ERPs produced by Seen and Unseen conditions under this paradigm.

## Materials and methods

### Participants

Nineteen volunteers participated in this experiment. Four of them were excluded from electrophysiological analysis, two because of excessive electric artifacts and two because they had a false positive rate above a predefined threshold (see Visual Stimulation below). In total, 15 subjects (8 females) were used for analysis. Ages ranged from 22 to 33 years (26.5 ± 3.5; mean ± SD). Every participant had normal or corrected to normal vision. This work was approved by the ethics committee of the Escuela de Psicología, Pontificia Universidad Católica de Chile and was conducted accordingly to its guidelines. Written informed consent was given by all participants prior to the experiment.

### Visual stimulation

**Figure 1:**
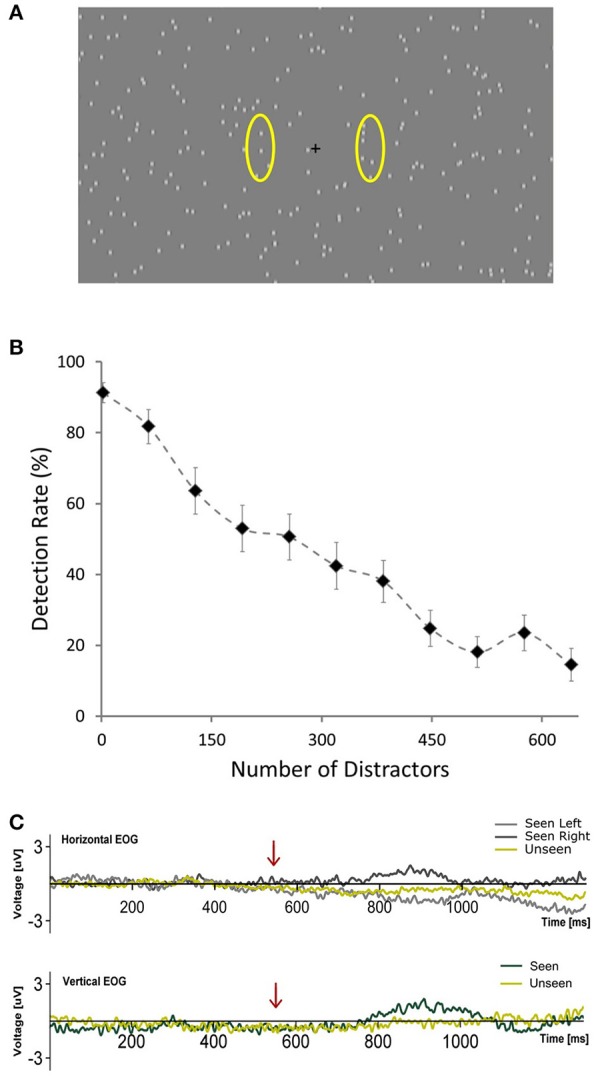
**(A)** Depiction of the general scene of the visual stimulation showing the central fixation cross and distractors placed randomly across the screen. Yellow ovals (not presented during the experiment) illustrate the approximate areas where targets occurred. (**B**) Plot showing the average percent of detected targets across subjects as a function of the number of distractors presented per frame during the calibration phase. Error bars represent SEM. (**C)** Horizontal and Vertical Electrooculograms for Seen and Unseen conditions averaged across subjects. Time zero corresponds to the beginning of the cue (blinking of the fixation cross). Arrows indicate the start of target presentation.

A general scheme of the visual stimulation is depicted in Figure 1A (220 distractors). It was presented on a computer screen (60 Hz refresh rate) at 60 cm from subjects. During the whole trial hundreds (see below) of distractors were continuously presented on the screen. They consisted of small (0.3°) gray dots, with blurred edges, each one continuously appearing and disappearing at random locations on every refresh of the screen (17 ms). This flickering pattern of distractors was present throughout the whole trial, including the period of target presentation. Targets consisted of a small dot, identical to a distractor, which moved downward at constant speed (11°/s) for 100 ms (6 frames). This occurred either at the left or right of the central fixation cross (6.25° of eccentricity). Subjects were given one button for each hand and were instructed to press the corresponding one accordingly to the side where they detected the target (Seen trials). With this we aimed at obtaining information not only whether they saw a target but where (objective performance). This way we could verify that subjects were responding specifically to the presented stimulus. They were asked not to press a button if they did not consciously perceive a target to simplify response requirements (Railo et al., 2015). Subjects were instructed to respond only when they were certain that they had consciously perceived the target rather than giving their responses as fast as possible. Subjects were instructed to remain visual fixation on the fixation cross at all time. No instruction regarding blinks was given. Each trial commenced with the blinking of the fixation cross (150 ms disappearance), followed, in 85% of trials, after 550 ms by a target. The 15% of catch trials without target presentation were introduced to assess target/distractor discriminability. This blinking cued subjects to when a target would be presented, but not about its location, which was randomized for each trial. Accordingly, trials were classified into one of three categories: Seen trials, in which subjects correctly reported both the presence and side of the target; Unseen trials, in which a target was presented but subjects failed to consciously perceive it. False positive trials, in which subjects either responded seeing a target when none was presented (catch trials) or when they incorrectly reported the side of presentation of the target. Two of the total 19 subjects presented more than 2.5% of False Positive trials, which indicated that they failed to sufficiently discriminate between targets and distractors. They were excluded from further analyses. We considered a group of 8 trials as a block. A 2.8 ± 0.2 s period with distractors and fixation cross but without cue or target was delivered after each block to avoid habituation. Every 10 blocks a resting period of 45 s with just a gray screen was allowed. Subjects could voluntarily shorten the duration of these resting periods. The whole experiment consisted of 75 blocks, totaling 600 trials per subject. The first 21 blocks were used to calibrate detection rate (see below). The duration of the entire experiment ranged between 30 and 35 min. All visual stimuli were programed using Psychopy (Peirce, 2007).

### Calibration phase

Pilot experiments showed that target detection rate was strongly dependent on the number of distractors per screen, with considerable inter-subject variability. In order to ensure statistical comparability between conditions we were interested in having a similar number of seen and unseen trials. Accordingly, we implemented a calibration phase to estimate the number of distractors that, for each subject, would generate a detection rate of ~50%. During the first 21 blocks of the experiment (168 trials, calibration phase) the number of distractors changed, increasing linearly from 2 in the 1st block, to 640 in the 11th block and then decreasing linearly back to 2 in the 21th block. At the end of the 21th block an algorithm calculated online the detection rate as the ratio between seen trials and non-catch trials for each block. It then fitted this data to a logistic equation: y = 1/(1 + e∧((x-b)/32)), where y is the detection rate, x is the number of distractors presented and b is the inflection point of the logistic curve, i.e., the theoretical amount of distractors necessary to yield a 50% detection rate. The value of b that best fitted the subject's calibration phase data was the number of distractors used for the remainder of the experiment for that subject. Electrophysiological data from the calibration phase were not analyzed.

### EEG data acquisition and analysis

Ongoing electrical brain activity was measured using a digital 32-electrode (Ag-AgCl) EEG system (Biosemi ActiveTwo, 24 bits, 2048 Hz sampling frequency) complemented by six additional electrodes. Two mastoid reference electrodes and 4 electrooculogram (EOG) electrodes were placed above and below the right eye and on the outer canthi of each eye. Vertical and Horizontal EOGs were obtained by means of bipolar derivations of the corresponding electrodes (Figure 1C). Original recording reference followed standard Biosemi DRL/CMS procedure. Afterwards, scalp electrodes data were offline re-referenced to the average of the two mastoid electrodes for every analysis unless stated otherwise (e.g., **Figure 5**, bottom). Analyses were made using MATLAB (The MathWorks, Inc. USA) toolboxes EEGLAB (Delorme and Makeig, 2004) and ERPLAB (Lopez-Calderon and Luck, 2014) in addition to custom made scripts. Raw continuous EEG data were filtered between 0.1 and 100 Hz (Butterworth filter, 4th order, forward and backward to avoid phase artifacts) and segmented in 2.5 s periods starting 0.5 s before the beginning of the cue. We used a moving window peak-to-peak threshold criterion (threshold = 200 uV, window size = 200 ms, step = 100 ms) to automatically reject artifact-containing trials. Trials were then visually inspected in order to discard any trials with remaining eye-movement related artifacts that could have gone undetected by the automatic rejection procedure. In average, 28.6% (*SD* = 19.8%) of the trials were rejected across subjects. An average of 129.6 trials for each subject were used in subsequent analyses for each condition (Hit = 128.1, Miss = 131.2).

**Figure 2:**
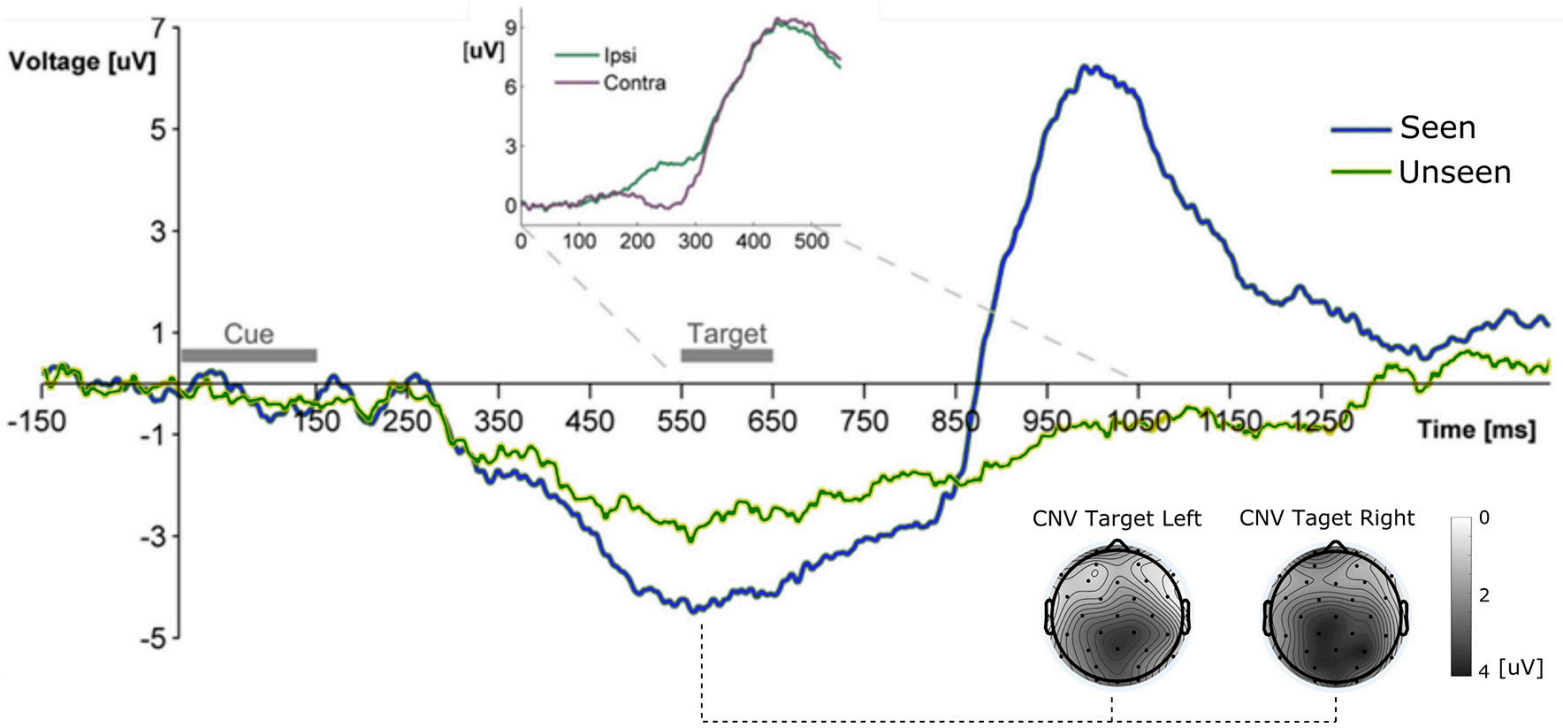
Grand Average ERP waveform showing the mean voltage recorded from Pz electrode across subjects for Seen and Unseen conditions. Two main components are observable, a negative deflection (CNV, topoplots at bottom right for left and right seen targets) peaking right before target presentation and a latter target-evoked P3b component, only present for Seen trials. The Inset shows the ERP waveform evoked by the target during Hit trials from parietal electrodes (P3/P4) ipsilateral and contralateral to the visual hemifield where the target appeared. The difference between both curves corresponds to the N2pc component.

**Figure 3:**
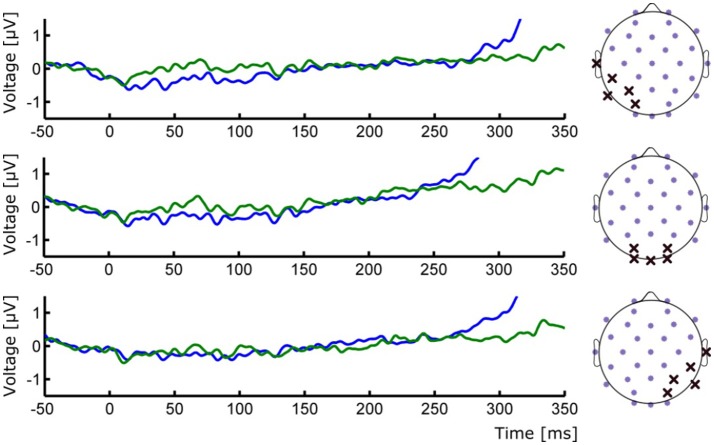
(**Left**) ERP waveforms of occipital ROIs depicting the absence of early P1/N1 components. (**Right**) Positions of the selected electrodes for each corresponding ROI. Group averages for Seen (blue line) and Unseen (green line) conditions are shown.

**Figure 4:**
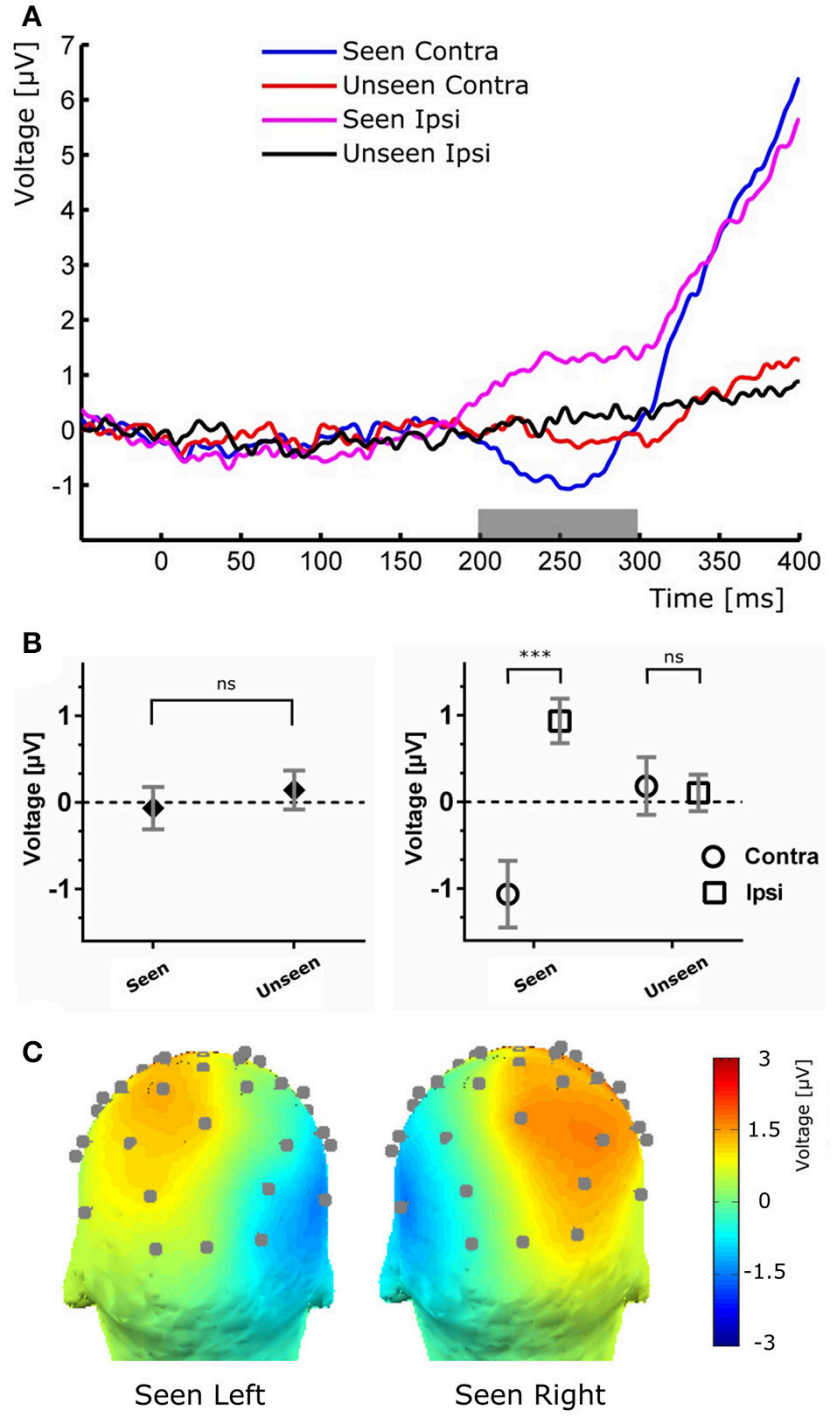
**(A)** Target-evoked ERP waveform showing the N2pc component as the difference between ipsilateral and contralateral electrodes. **(B)** Quantification of the N2pc component without (left) and with (right) the ipsilateral and contralateral distinction for the same time range. **(C)**, Scalp maps of Seen trials in which the target was presented at either the left (left) or right (right) of the fixation cross for the N2pc time range.

**Figure 5:**
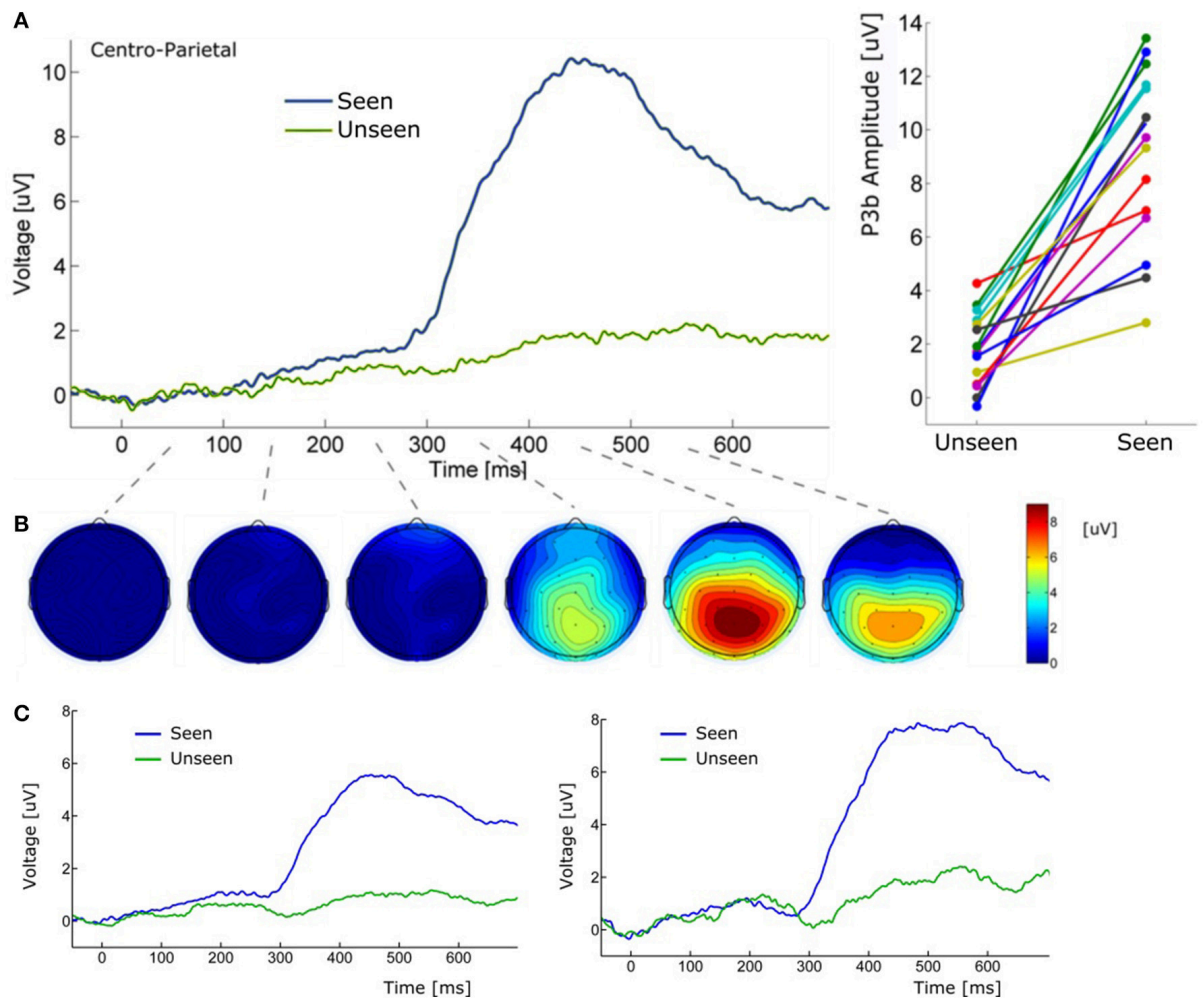
(**A)** (Left) Grand average ERP waveform (Pz electrode) for Seen and Unseen conditions. It shows that the P3b component was mainly evoked when subjects consciously perceived the target. (Right) Voltage amplitude for Seen and Unseen conditions in the P3b peak time range presented individually for each subject. (**B)** Scalp maps depicting the mean voltage difference between Seen and Unseen conditions. Each scalp map corresponds to successive non-overlapping 100 ms windows starting at time zero. **(C)** (Left) Grand average ERP of Pz electrode referenced to the average of all 32 scalp electrodes. (Right) Grand average ERP of Pz electrode referenced to a single frontal electrode (Fz).

**Figure 6:**
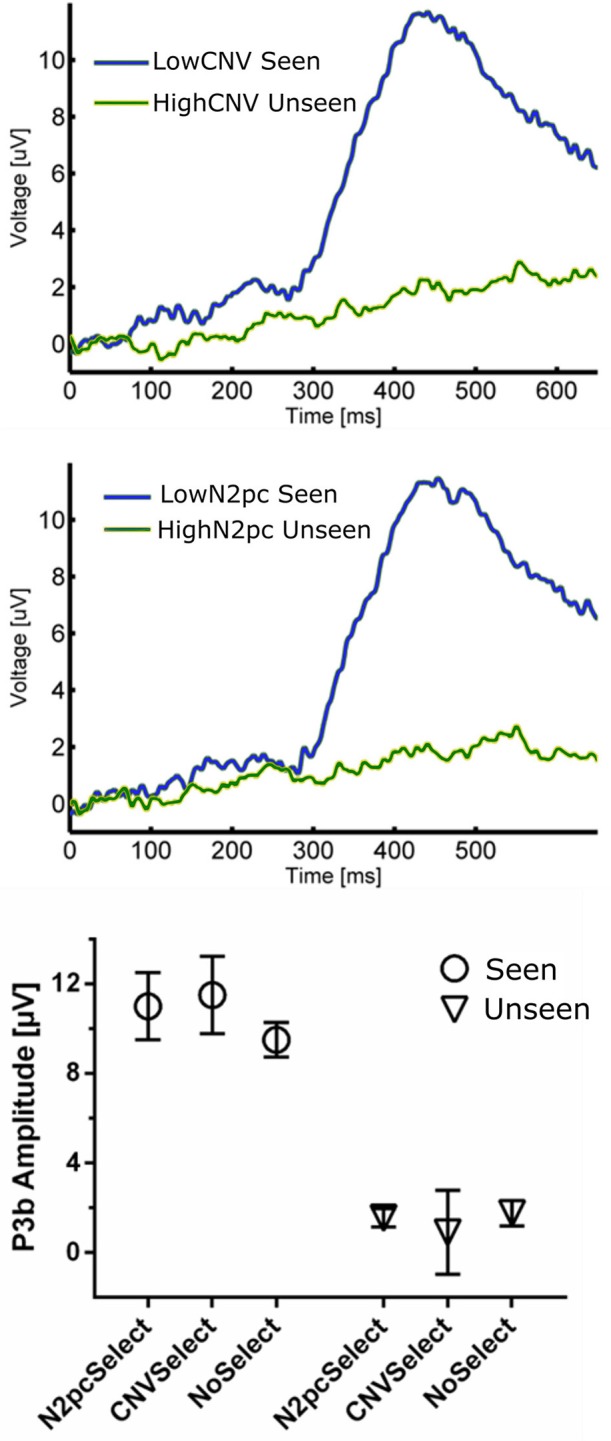
**(Top)** ERP waveforms constructed using CNV-selected and N2pc-selected Seen and Unseen Trials. It shows that P3b was evoked indistinctly of the trial selection procedure. (**Bottom**) P3b quantification of trial-selected and regular ERP waveforms. P3b's amplitude was unaffected by both trial-selection procedures.

For the construction of ERP waveforms, artifact-free trials from each subject were baseline corrected and averaged for each condition and afterwards across subjects to obtain the grand average waveform. No downsampling, smoothing or additional filtering was conducted on the data. Baseline correction periods were: a 200 ms period immediately preceding the cue for the general ERP waveform (Figure 2) and a 50 ms window immediately preceding target presentation for the target-evoked ERP waveform (Figures 3–**6**). Scalp maps depict the mean voltage of the grand average waveform in a defined time period, and were constructed using EEGLAB/ERPLAB toolboxes. For the exploratory analysis of early (<200 ms) ERPs we selected three ROIs (see Figure 3) and analyzed the statistical differences between conditions and between each condition and zero in 8 non-overlapping 25 ms time windows using Wilcoxon signed rank test. No correction for multiple comparisons was made. ERP quantification of each component was done using the mean amplitude in a time window. These time windows were: 400–500 ms after target presentation for P3b (Pz electrode), 200–300 ms for N2pc (P3 and P4 electrodes, see below) and 525–575 ms after the start of the cue for CNV (Pz electrode). For N2pc quantification we first obtained the mean voltage from electrodes P3 (left parietal) and P4 (right parietal) separately for targets that were presented either on the left or right. N2pc was quantified as the difference between the mean voltages of the contralateral electrode and the ipsilateral electrode (P3 or P4 depending on which side the target was presented). Finally we assessed possible relations between attention-related components, such as CNV and N2pc, to P3b. To do this we constructed two new waveforms using only half of the trials, which were selected according to the following procedure: for each subject we chose trials based solely on whether the single-trial amplitude of the CNV or N2pc fell in the upper or lower half of their corresponding total amplitude distributions, i.e., single trial amplitude was higher or lower than their corresponding median (High CNV, Low CNV, High N2pc, and Low N2pc, see section Results and **Figure 6**).

## Results

### Behavioral results

Figure 1B shows the relationship between number of distractors and detection rate across subjects during the calibration phase (first 168 trials), illustrating the marked effect that the number of distractors had on detectability. This correlation was observable on the single-subject level: 14 out of 15 subjects showed a significant correlation (Pearson's correlation) between the number of distractors and their detection rates that resisted Bonferroni correction (*r*^2^ across subjects = 0.80 ± 0.09, mean ± SD). At the end of the calibration phase, for each subject, the relationship between number of distractors and detection rate was modeled online using a logistic equation. With this we obtained an estimate of the number of distractors that would be expected to produce a 50% detection rate for each subject (see section Materials and Methods, Calibration Phase). This number varied across subjects from a minimum of 97 to a maximum of 577 distractors. The mean detection rate across subjects after the calibration phase was 57.4% ± 15.4 (mean ± SD) and was not different for left vs. right targets (*p* > 0.05, Wilcoxon signed rank test). Detection performance was not correlated (Pearson's correlation) with the number of distractors presented across subjects (number of distractors after calibration was 277 ± 154, mean ± SD; *r*^2^ = 0.017, *p* = 0.66). This supports the effectiveness of the calibration procedure. Additionally, subjects did not show a consistent learning effect, as revealed by a lack of correlation between the detection rate and the ordinal block number (mean *r*^2^ = 0.069, only one subject showed significant correlation after Bonferroni correction). Reaction time in the post-calibration phase was 620 ± 83 ms (mean ± SD). To ensure that subjects responded specifically to the presented targets we analyzed false positive trials, defined as either having a “seen” response in trials without a target (15%) or when subjects erroneously reported the side of presentation. False positive rates were very low (0.52 ± 0.62%; mean ± SD) in comparison with what would be expected from random behavior (57.5%). This confirms that subjects were able to specifically discriminate between targets and distractors.

### ERP results

#### General ERP waveform

All ERP measurements were done re-referencing data to the average of the two mastoid electrodes unless specifically stated (see section Materials and Methods). To study the broad electrophysiological behavior produced by our stimulation paradigm we constructed the grand mean across participants, shown in Figure 2. Shortly after the cue, and before target presentation, a prominent negative deflection was observed for both conditions. We characterize this component as a Contingency Negative Variation (CNV), which is related to the buildup of temporal expectation (Tecce, 1972; Nagai et al., 2004). CNV magnitude was significantly greater for Seen than for Unseen trials (*p* < 0.05, Wilcoxon signed rank test). Topoplots in Figure 2 show a somewhat posterior topological distribution of CNV around parietal electrodes and the absence lateralization of this component related to target side of appearance. Visual stimulation was identical for Seen and Unseen trials, so the observed differences in CNV amplitude suggests a relationship between whether a target was detected or not and the previous expectancy state of the subject. This expectancy state was probably enhanced by the fixed cue-stimulus latency, which was chosen to reduce the difficulty of the task while maintaining a detection rate around 50%. Five hundred and fifty milliseconds after the start of the cue the target was presented. No early (<200 ms) ERP components to the target were detectable (Figure 3). Two ERP components were evoked in response to target presentation: a difference between posterior contralateral- vs. ipsilateral-to-target electrodes in the 200–300 ms range, consistent with an N2pc (inset in Figures 2, 4) and broad parieto-central positivity consistent with a P3b (Figure 5), both strongly associated with conscious perception.

#### Target-evoked ERP components

To study the ERP components produced by the presentation of the target without interference from the ongoing CNV, in addition to the original baseline at the beginning of the trial, we baseline corrected the data with respect to the period immediately preceding target presentation. For a more stringent examination of early (<200 ms) ERP components we averaged electrodes into three regions of interest (ROIs): two lateral occipito-temporal ones and one central-occipital ROIs. Figure 3 shows the specific electrodes of these ROIs. Figure 3 also shows the absence of ERP components in the first 200 ms after target presentation. We tested whether Seen and Unseen conditions were statistically different from each other and from zero (independently) using 8 non-overlapping 25 ms time windows starting at the time of target presentation. Statistical Tests did not show significant differences for any comparison (all *p* > 0.05, Wilcoxon signed rank test, uncorrected). We also carried out this analysis in data re-referenced to the average of all scalp electrodes and found the same results (all *p* > 0.05, Wilcoxon signed rank test, uncorrected). Thus, Seen and Unseen conditions showed no difference from each other or from zero in the first 200 ms after target presentation. Only after 200 ms target-evoked components started to appear. Although not evident initially (see inset in Figures 2, 4B, left), the first target-evoked component we observed was a voltage difference between posterior contralateral and ipsilateral electrodes occurring from 200 to 300 ms after target presentation (Figure 4A). This is consistent with an N2pc, an ERP related to the automatic and lateralized shifting of visuospatial attention (Luck and Hillyard, 1994; Anllo-vento et al., 1998; van Velzen and Eimer, 2003; Robitaille and Jolicoeur, 2006; Hickey et al., 2009). To further examine this component we conducted a two-way ANOVA for its amplitude (lateralization and perception as factors) and found a strong main effect of lateralization (Contralateral vs. Ipsilateral; *p* < 0.007, *F* = 10.01) but not of the perceptual factor (Seen vs. Unseen; *p* > 0.05; *F* = 0.47). We also found a significant interaction effect (*p* < 10^−3^; *F* = 19.3, see Figure 4B, right panel). Sydak's multiple comparison test revealed that contralateral and ipsilateral conditions were different from each other for Seen trials (*p* < 10^−3^) but not for Unseen trials (*p* > 0.05). This means that the N2pc occurred with significant amplitude only when subjects consciously perceived the target. As expected for this component, scalp maps showed a negative potential over occipital electrodes contralateral to where the target was detected (Figure 4C). No other differential behavior between contralateral and ipsilateral sites was found for any other time window.

About 300 ms after target presentation a strong positive deflection was observed over centro-parietal electrodes (Figures 5A,B). Because of its latency and scalp localization, we characterize this potential as a P3b (Polich, 2007). As with N2pc, P3b's magnitude showed robust differences between Seen and Unseen trials (*p* < 10^−5^ Wilcoxon signed rank test). Moreover, P3b was markedly reduced in unseen trials (Figure 5), although its amplitude resulted significantly different from zero (*p* < 0.05, Wilcoxon signed rank test). Importantly, P3b's association with conscious perception consistently appeared at the single subject level. This can be seen in Figure 5A (right side) which shows the mean P3b amplitude, for each subject, for Seen and Unseen trials. Although there is considerable inter-subject amplitude variability as expected for scalp EEG studies, every subject systematically presented higher mean P3b amplitude when they consciously perceived the target compared to when they did not. As mentioned above, a number of reports have described VAN specifically associated with conscious perception (Koivisto et al., 2008; Pitts et al., 2011; Harris et al., 2013; Rutiku et al., 2016). We therefore compared seen vs. unseen contralateral-to-stimulus electrode amplitudes in the 200–300 ms range. We did not find statistically significant differences either in P3/P4 (*p* = 0.18), P7/P8 (*p* = 0.218), or PO3/PO4 (*p* = 0.538), Wilcoxon signed rank test, uncorrected). The abovementioned ERP waveforms used linked mastoid electrodes for re-referencing data, which are located near areas in which VAN has been shown to have strong amplitude. As a precaution, we therefore reanalyzed the data using two different referencing methods. Figure 5C shows target-evoked ERP waveforms with a single (monopolar) frontal electrode (Fz) reference and using 32-electrodes average referencing. No qualitative difference is observable in comparison to mastoid-referenced data.

#### Independence between P3b, CNV, and N2pc

The relationship between P3b and conscious perception has been illustrated with various experimental strategies (Koivisto and Revonsuo, 2010). N2pc on the other hand, has been related to focusing attention on a portion of the visual field (Luck and Hillyard, 1994; Robitaille and Jolicoeur, 2006) and, to our knowledge, it has not been linked to conscious perception. To assess whether these two components reflect distinct underlying cognitive processes, we studied their possible relationship within our data. We also compared these components to the CNV, which also has an attentional role, albeit a different one than N2pc (Prescott and Andrews, 1984; Van Rijn et al., 2011). Specifically, to test the possible relation between P3b and these two attentional ERPs we used two complementary strategies: First, we computed the correlation between the mean P3b amplitude and both CNV and N2pc amplitudes across subjects. This was done using the magnitude of these components evoked in Seen trials, as N2pc was not present for Unseen trials (Figure 4). We found no significant correlation, either for CNV (*r*^2^ = 0.076, *p* = 0.32) or for N2pc (*r*^2^ = 0.00026, *p* = 0.95; Pearson's correlations). Second, we employed a trial-selection strategy: For each subject we selected half of the total trials accordingly to whether the N2pc and CNV single trial amplitude was above or below the median (i.e., High N2pc, Low CNV, etc.). In particular, because attention has been proposed to be necessary for conscious perception (Meuwese et al., 2013) (but see Wyart and Tallon-baudry, 2008), we analyzed ERP waveforms of seen trials with low attentional components (Low CNV/N2pc) and unseen trials with high attentional components (High CNV/N2pc). With these CNV-selected and N2pc-selected waveforms we compared the resulting P3b amplitude across conditions (Figure 6). Two-way ANOVA on P3b's amplitude with perception (Seen-Unseen) and trial selection (CNV/N2pc-selection) as factors showed no global effect. Trial selection had no significant effect on P3b's magnitude (Figure 6, trial selection factor *p* > 0.05, *F* = 1.29). P3b continued to exhibit systematically higher mean amplitude in Seen trials (Perceptual factor, *p* < 10^−4^, *F* = 58.54), and showed no magnitude modulation by either CNV or N2pc components (Sydak's multiple comparisons, all *p* > 0.05). This result indicates that P3b's magnitude is not correlated with CNV or N2pc's amplitudes, under both subject's average and single trial analysis. This is consistent with the contention that, in our experimental paradigm, P3b is related to different cognitive processes than those underlying CNV and N2pc.

## Discussion

In the present study we explored the electrophysiological activity associated to the process of conscious perception. The novelty that we introduce here, in addition to modern standards for the study of consciousness such as physical equality between conditions, is the dynamic nature of the object of such conscious perception. Distractors and targets differed only by virtue of their dynamic behavior (cf. flickering v/s transient but consistent motion), while their static features like shape, size, contrast, etc. were the same. This way, we isolated movement as the main feature that subjects consciously perceived. Following target presentation, no ERP component was evoked before 200 ms, regardless whether the target was detected or not. In contrast, two later ERP components, N2pc and P3b, were differentially evoked between Seen and Unseen conditions.

It could be argued that the absence of early (<200 ms) ERPs in our data is the result of insufficient number of trials per condition. However, this is unlikely because robust P1 modulations have been shown using similar (Roeber et al., 2008) and even less (Veser et al., 2008) number of trials. Although components like P1 has been previously proposed as an NCC (Pins and Ffytche, 2003; Roeber et al., 2008; Veser et al., 2008), alternative accounts for its modulations in some NCC experiments point toward circumstantial attentional differences rather than to it constituting a proper NCC (Koivisto et al., 2008; Koivisto and Revonsuo, 2010). The present work is not the first to report a lack of early ERPs in the search of visual NCCs. Previous examples include works by Busch and VanRullen (2010b) using very small static stimuli and that of Rutiku et al. (2016) using a great diversity of static stimuli. Both studies show no early ERP components. It is likely that the small size and mild contrast difference associated with target presentation could have contributed to the lack of early ERP components we observed (Figure 3). Experimental paradigms that produce P1/N1 activity in motion perception normally use much bigger and higher contrast stimuli (Bach and Ullrich, 1994; Niedeggen et al., 2004, 2015). However, as evidenced by the behavioral results, and despite the demanding perceptual and attentional conditions of our task, conscious perception of motion was consistently achieved in “Seen” trials. This strongly suggests that P1 and reliable conscious perception (here supported by the extremely low false positive rate) are not required to occur concomitantly in visual perception paradigms. This does not preclude the possibility of early components as content-specific NCCs for other type of stimuli; however, our data show no evocation or correlation of this component to conscious perception of the particular content used here.

Cortical activity caused by visual stimulation, including that specifically evoked by motion, has been found initially in the primary visual cortex (Tootell et al., 1995; Andersen, 1997; Culham et al., 2001). Neural activity then segregates into two visual streams, ventral and dorsal, each one composed of different cortical areas with divergent receptive fields (Goodale and Milner, 1992; Milner and Goodale, 2008). Shape-like information is believed to preferentially activate the ventral stream while dynamic patterns like movement or expansion/contraction of an object (approaching/retreating) preferentially activate areas in the dorsal stream (Van Essen and Manusell, 1983; Goodale and Milner, 1992; Milner and Goodale, 2008). In this line, it is possible that, compared to classical stimuli, the stimuli used here produced a predominant activation of dorsal pathways. This contrasts with static images like faces or numbers, which preferentially, but not exclusively, activate the ventral visual stream. Importantly, this could be an alternative explanation as to why we do not observe the (typically occipito-temporal) VAN component (Figures 3–5). Indeed, activation of specific areas of the visual system can generate specific observable ERP components. This is the case for the N170 component, which is produced by activation of particular regions in the ventral stream such as the fusiform face area (Nguyen and Cunnington, 2014). Nevertheless, to fully discern VAN's role in conscious motion perception, further experimentation contrasting different dynamic and static visual stimulus is required (see also below).

In the 200–300 ms time period we observed a posterior lateralization of the ERP waveform: both a negative deflection over contralateral regions and a positive one in ipsilateral scalp electrodes in the Seen condition. Although we interpret this as an N2pc component (Figure 4), it is worth mentioning that the contralateral negativity for Seen trials, although not statistically significant in our data, could be interpreted as evidence of an underlying VAN, which could be contributing to the overall N2pc effect. N2pc has long been ascribed to cognitive processes associated with the spatial focusing of visual attention (Luck and Hillyard, 1994; Eimer, 1996). It can be observed in visual search settings in which subjects must discriminate between a target and distractors that appear at unpredictable locations (Robitaille and Jolicoeur, 2006), as in our task. Although N2pc only occurred with significant amplitude for Seen trials, there is evidence in the literature against N2pc as a proper NCC (but see Crouzet et al., 2017). Its amplitude is indeed unaffected by visual masking that abolish conscious perception (Woodman and Luck, 2003; Robitaille and Jolicoeur, 2006; Woodman, 2010). In particular, when conscious perception of a stimulus is impeded by 4-dot masking (Enns and Di Lollo, 1997), N2pc's amplitude remains the same for masked and unmasked stimulus (Robitaille and Jolicoeur, 2006). Additionally, it has been shown in a series of elegant priming experiments that when both prime and target have a lateralization toward the same hemifield, only the prime, but not the target, produces an N2pc, even when the target but not the prime is consciously perceived (Jaśkowski et al., 2002, 2003). Although our experimental paradigm cannot fully discern N2pc's role in conscious perception, exploring its relation to P3b did produce some important insights: as with the CNV evoked by the cue, we found no correlation between N2pc amplitude and P3b amplitude, either at the subject or at the single trial level (Figure 6). In the context of previous studies that have associated N2pc with automatic lateralized shifts of attention (Tecce, 1972; Delorme and Makeig, 2004; Nagai et al., 2004; Lopez-Calderon and Luck, 2014), these results are consistent with it being a prerequisite rather than a proper NCC.

In our experiment we used a fixed cue-stimulus time interval. This lead to the elicitation of a CNV (Trillenberg et al., 2000), which was differentially evoked between conditions, being in average stronger prior to the detection of a target when compared to missed targets. Because Seen trials and Unseen targets were classified as such only after subjects' response, this suggests that subjects were more likely to detect target stimuli when the buildup of expectation was greater in the preceding period. The distribution of CNV in our data is somewhat more posterior compared to other studies, peaking around Pz electrode instead Cz electrode (Trillenberg et al., 2000). A possible reason for this could be that subjects were not instructed to respond as fast as possible, nor was our task a forced choice task, as is the case for the majority of comparable studies, which perhaps diminished the motor preparation requirements. Nevertheless further research is required to elucidate this point. CNV's presence could be interpreted as a limitation of the present study as the presence of CNV could theoretically have influenced our ability to observe early ERP components, as they would overlap. However, we believe this to be unlikely because: (1) we were able to observe N2pc regardless of its overlap with CNV, (2) P1/N1 complex has been observed overlapping with CNVs in other paradigms (Hasler et al., 2016), and (3) CNV is a much slower component as compared to, for example, the P1/N1 complex and is produced by different cortical areas.

P3b showed strong amplitude modulations by conscious perception in our study, regardless of the re-referencing method (Figure 5). This component has been systematically proposed as a proper NCC in the literature (Sergent and Dehaene, 2004; Del Cul et al., 2007; Koivisto and Revonsuo, 2010; Dehaene and Changeux, 2011). Evidence for this comes from experiments that use different strategies to assess NCCs, such as masking (Rutiku et al., 2015), change blindness (Busch et al., 2010a), binocular rivalry (Veser et al., 2008), degraded stimuli (Melloni et al., 2011), as well as in non-visual sensory modalities such as the auditory modality (Bekinschtein et al., 2009), among others. In these experiments, alongside with P3b modulations, usually other components like VAN or P1 also show amplitude modulations associated with conscious perception. Here we show P3b modulations without concomitant P1 or VAN modulations during the conscious perception of motion. This suggests independence between the cognitive processes behind P3b and those behind P1 and VAN. Our results are therefore consistent with the contention that, regardless of the static or dynamic nature of the stimulus, conscious perception of relevant stimuli appears to consistently produce P3b as a neural signature.

Some paradigms, however, have shown no P3b amplitude difference between Seen and Unseen conditions (Koch et al., 2016). Recent work by Pitts et al. (2014) has suggested an alternative explanation for the systematic occurrence of P3b in NCC experiments. In their experiments subjects were presented with two types of stimuli, and were asked to respond only when they detected one of the two types. This effectively made one kind of stimuli relevant and the other irrelevant. In addition to VAN, and in agreement with our results, when subjects perceived the relevant stimuli a strong P3b was evoked. But when subjects were presented with irrelevant stimuli, which were nevertheless reported as perceived, no P3b was observed. However, VAN still distinguished Seen from Unseen conditions. The authors' interpretation is that P3b would be related to post-perceptual processing of relevant stimuli, but not to conscious perception *per se* which would be more tightly correlated to the neural processes underlying the VAN. However, as the authors themselves acknowledge, the way conscious perception was assessed makes their results difficult to interpret. Subjects responded a questionnaire regarding whether they had seen the irrelevant stimuli at the end of a 600 trials block. Their answers were therefore most probably a generalization of previous experiences based on the memory of something that (1) was irrelevant for them and (2) had occurred in average more than 5 min ago. This contrasts with traditional NCC experiments were the answer is given almost immediately and on a trial-by-trial basis, as was the case in the present work. One could speculate about alternative explanations as to why P3b is not observable in response to conscious perception of irrelevant stimuli. In the terms of Shadlen and Kiani (Shadlen and Kiani, 2011; Dehaene et al., 2014), conscious perception of a stimuli is reached when the system “decides to engage” with it. The lack of urgency toward the irrelevant stimuli could induce that subjects do not consciously perceive the stimuli as fast as possible. Accordingly, when a stimulus is rendered irrelevant, if the system engages with it at all, it will do so only when sufficient resources are available. This would result in an increase in the temporal variability between the presentation of irrelevant stimuli and when the subject becomes conscious of it. This is in line with recent work by Sergent et al. (2011, 2013) that show that there can be a temporal dissociation between when a stimulus is consciously perceived and when it is presented. Such variability would disrupt the time locking of the underlying neuronal process required for a P3b to be observed (Luck, 2014). If this was the case, it could explain why Pitts et al. (2014) found no P3b associated with conscious perception of irrelevant stimuli. It is worth noting that other experimental strategies directly manipulating stimulus relevance while studying visual NCCs, have found results consistent with P3b as a proper NCC (Rutiku et al., 2015). However, expectations have also been found to influence P3b's correlation with conscious perception, as shown by the work of Melloni et al. (2011) using a more standard report strategy. They found that when the target stimulus is highly expectable, P3b ceases to correlate with conscious perception. In general these evidences indicate that context or particular situations influence the neural signatures of consciousness. In particular, manipulation of both relevance and expectations appear to modulate the P3b evoked by conscious perception, which would thus indicate that P3b is a context-dependent NCC.

In summary, our results show that motion, as well as several static visual stimuli (Kim and Blake, 2005), differentially evoke the P3b component in the context of a relevant and partially unpredictable (here in terms of location) target stimuli. Thus our data support the role of P3b as a content-independent albeit not completely general (context-independent) NCC, while highlighting the importance of prerequisites as evidenced by the N2pc and CNV results. Further experimentation exploring specifically the effects potential modulators such as context or relevance, are required to advance in disentangling the neuronal mechanisms underlying the conscious perception of motion.

## Author contributions

DC and GB designed the experiment. GB conducted the experiments and analyzed the data. DC and GB wrote the manuscript.

### Conflict of interest statement

The authors declare that the research was conducted in the absence of any commercial or financial relationships that could be construed as a potential conflict of interest.
